# Effect on the Antioxidant, Lipoperoxyl Radical Scavenger Capacity, Nutritional, Sensory and Microbiological Traits of an Ovine Stretched Cheese Produced with Grape Pomace Powder Addition

**DOI:** 10.3390/antiox10020306

**Published:** 2021-02-17

**Authors:** Raimondo Gaglio, Ignazio Restivo, Marcella Barbera, Pietro Barbaccia, Marialetizia Ponte, Luisa Tesoriere, Adriana Bonanno, Alessandro Attanzio, Antonino Di Grigoli, Nicola Francesca, Giancarlo Moschetti, Luca Settanni

**Affiliations:** 1 Dipartimento Scienze Agrarie, Alimentari e Forestali, Ed. 5, Università degli Studi di Palermo, Viale delle Scienze, 90128 Palermo, Italy; raimondo.gaglio@unipa.it (R.G.); marcella.barbera@unipa.it (M.B.); pietro.barbaccia@unipa.it (P.B.); marialetizia.ponte@unipa.it (M.P.); adriana.bonanno@unipa.it (A.B.); antonino.digrigoli@unipa.it (A.D.G.); nicola.francesca@unipa.it (N.F.); giancarlo.moschetti@unipa.it (G.M.); 2 Dipartimento di Scienze e Tecnologie Biologiche, Chimiche e Farmaceutiche, Università degli Studi di Palermo, Via Archirafi 34, 90123 Palermo, Italy; ignazio.restivo@unipa.it (I.R.); luisa.tesoriere@unipa.it (L.T.); alessandro.attanzio@unipa.it (A.A.)

**Keywords:** antioxidants, grape pomace powder, *Lactococcus lactis*, lipoperoxyl radical scavenger capacity, ovine stretched cheese, polyphenols, volatile organic compounds

## Abstract

An innovative ovine cheese enriched with red grape pomace powder (GPP) was produced to improve the functional properties of Vastedda cheese typology. Vastedda cheese making was performed adding GPP and four selected *Lactococcus lactis* strains (Mise36, Mise94, Mise169 and Mise190). For each strain, 40 L of pasteurized ewe’s milk was divided into two aliquots representing control and experimental trials. Control cheese (CC) production did not contain GPP, while the experimental cheese (EC) production was enriched with 1% (*w*/*w*) GPP. GPP did not slow down starter development and acid generation. Plate counts and randomly amplified polymorphic DNA (RAPD)-PCR analysis confirmed the dominance of the starters in all trials. The evolution of the physicochemical parameters showed that EC productions were characterized by lower fat content, higher protein content, and higher values of secondary lipid oxidation. Sensory evaluation indicated that the cheeses produced with the strain Mise94 were those more appreciated by the judges. Thus, the last cheeses were investigated for some functional aspects: GPP enrichment significantly increased antioxidant activity and lipoperoxyl radical scavenger capacity, confirming that grape polyphenol inclusion in cheese represents an optimal strategy for the valorization of ovine cheeses as well as winemaking industry by-products.

## 1. Introduction

Nowadays, natural antioxidants extracted from plant by-products represent a useful source of active compounds to produce healthy and functional foods more and more requested by consumers [1]. In order to face this increasing demand, in the last years, the food industries also focused the attention toward dairy products. Thus, several efforts are being made to enrich dairy products such as cheeses with functional and antioxidant components [2] because these foods are basically poor in bioactive compounds [3].

The most interesting natural antioxidants belong to the chemical classes of phenols [4]. They are particularly present in some fruits such as red grape [5]. Thus, winemaking industry by-products constitute an interesting source of bioactive phenolic compounds [6]. The main by-products generated during the winemaking process are composed by a mix of grape seeds and skins [7], namely grape pomace, which represents approximately 20–25% of the grapes at harvest [8]. The wine industry produces millions of tons of grape pomace with several environmental and economic implications [9]. These by-products are characterized by high contents of antioxidant polyphenols and dietary fiber with potential beneficial effects on human health, including antioxidant activity and antimicrobial, anti-inflammatory, anticancer, and cardiovascular properties [10].

Grape pomace in powder (GPP) has been added to several food formulations including cereal-based foods [11], meat [12], and fish [13] products as well as cheeses [7,14] in order to increase their antioxidant activity and dietary fiber content. Regarding GPP addition to cheese, so far, this application has only been performed for bovine milk derived cheeses.

In Italy, dairy sheep farming is particularly located in the islands of Sardinia and Sicily and in central regions. Sheep milk produced in Sicily derives mainly from ewes of Valle del Belice and Comisana local breeds fed pasture-based diets and is processed to manufacture dairy products at industrial or farm level. Sicily region valorized some of its dairy productions by means of the recognition of quality status like Protected Designation of Origin (PDO), but due to their scarce reputation in terms of healthy properties, cheeses, especially those processed from ewe’s milk, are considered as unsuitable for a valuable nutrition by the majority of people. For this reason, in the last years, several breeders and cheese producers pushed research institutes to develop dairy functional foods in order to contribute to the rural development. This issue is particularly relevant for the Mediterranean area because the production of cheeses is important for the environmental sustainability, makes advantageous the maintenance of native breeds, allows the survival of handicraft techniques, limits the land abandonment phenomenon, and a renewed interest for high-quality, safe products can increase the employment for livestock raising and milk transformation and have an appreciable impact on the local economy.

With this in mind, in this work a novel fresh-stretched ewes’ milk cheese was produced with the addition of GPP using four lactic acid bacteria (LAB) strains individually. These LAB were previously selected for their resistance in presence of the main grape polyphenols [15]. The final cheeses were subjected to the evaluation of the microbiological, physicochemical, sensory, and functional aspects.

## 2. Materials and Methods

### 2.1. Grape Pomace Powder Production and Natural Milk Starter Culture Preparation

Grape pomace powder (GPP) was prepared from red grape pomaces of Nero d’Avola *cultivar* provided by the winemaking factory “Cantine Europa” located in Petrosino (TP, Sicily, Italy) following the methodology described by Marchiani et al. [7]. Grape pomaces were dried in the semi-industrial oven Compact Combi (Electrolux, Pordenone, Italy) at 54 °C for 48 h and milled through a Retsch apparatus (Haan, Germany) to a particle size of 250 μm.

Four different natural milk starter cultures (NMSCs) were developed with four strains, individually inoculated, of *Lactococcus lactis* (Mise36, Mise94, Mise169, and Mise190) belonging to the culture collection of the Department of Agricultural, Food, and Forest Sciences (University of Palermo, Italy). These strains were previously isolated from fermented raw ewes’ milks added with the main grape polyphenols commonly found in winery by-products and selected as technologically relevant for dairy applications [15]. All strains were cultivated in M17 broth (Biotec, Grosseto, Italy) at 30 °C for 24 h and centrifuged at 10,000× *g* for 5 min. The cells were then washed twice in Ringer’s solution (Sigma-Aldrich, Milan, Italy) and re-suspended in the same solution. The washed cells of each LAB strain were singly inoculated (1%, *v*/*v*) into 1 L of whole fat UHT milk (Conad, Mantova, Italy) and incubated at 30 °C for 24 h when they reached a concentration of approximately 10^9^ colony forming units (CFU)/mL, as ascertained by plate count. These fermented milks represented the four NMSCs to be used in cheese production.

### 2.2. Cheese Production and Sample Collection

The experimental cheese making trials were carried out at a dairy pilot plant (Biopek, Gibellina, Italy) following the protocol of production for the stretched cheese “Vastedda” [16]. The experimental plan included two different cheese productions for each LAB strain for a total of eight trials: CP36, control production with *L. lactis* MISE36; EP36, experimental production with *L. lactis* MISE36 + 1% of GPP; CP94, control production with *L. lactis* MISE94; EP94, experimental production with *L. lactis* MISE94 + 1% of GPP; CP169, control production with *L. lactis* MISE169; EP169, experimental production with *L. lactis* MISE169 + 1% of GPP; CP190, control production with *L. lactis* MISE190; EP190, experimental production with *L. lactis* MISE190 + 1% of GPP. Each trial was obtained with 20 L of pasteurized (72 °C for 15 s) ewes’ milk. Cheese productions were performed in plastic vats after sanitization with a solution of 2% (*v*/*v*) of H_2_O_2_ and acid adjuvant (PROMOX P900, Leggiuno, Italy) for 15 min to eliminate contaminant bacteria and fungi. Milk (cooled at 38 °C) was firstly inoculated with the corresponding NMSC (200 mL) to reach the final cell densities of 10^7^ CFU/mL and secondly added with 6 mL of liquid rennet (Fromase^®^ 220 TL, DSM Bright Science Brighter Living, Heerlen, Netherlands). After coagulation, the curds were broken with a stainless-steel curd beater until small rice-size grains were obtained. After whey draining, the control curds were immediately put into perforated containers while the experimental curds were added with 1% (*w*/*w*) of GPP, which was manually mixed and then transferred into perforated containers. The pH of control and experimental curds was monitored electrometrically by means of the pH-meter pH 70 + DHS (XS Instruments, Carpi, Italy). When curd acidification reached pH values in the range 5.20–5.40, the curds were stretched under hot (80–85 °C) water and molded into a bun shape. The cheeses were salted in saturated brine for 30 min, dried for 24 h at room temperature, packaged under vacuum, and kept under refrigeration (5 °C) for 15 d. All cheese trials were carried out in duplicate in two consecutive weeks. The sampling points, the number of samples analyzed, and the analyses performed are reported in Table 1.

### 2.3. Microbiological Analyses

Cell suspensions of milk samples were directly subjected to decimal serial dilutions in Ringer’s solution, while GPP, curd and cheese samples (15 g) were first homogenized with Ringer’s solution (135 mL) by the stomacher Bag-Mixer 400 (Interscience, Saint Nom, France) for 2 min at the maximum speed (blending power 4) and then 1:10 serially diluted.

Cell suspensions of GPP were used to enumerate the following microbial groups: Total mesophilic microorganisms (TMM) on plate count agar (PCA), after incubation at 30 °C for 72 h; LAB rods and cocci on de Man-Rogosa-Sharpe (MRS) agar and M17 agar, respectively, both incubated at 30 °C for 48 h in anaerobiosis; members of the *Enterobacteriaceae* family, detected on violet red bile glucose agar (VRBGA), after incubation at 37 °C for 24 h; yeasts on dichloran rose bengal chloramphenicol (DRBC) agar, incubated at 28 °C for 24 h; coagulase-positive and coagulase-negative staphylococci (CPS and CNS, respectively) on Baird Parker (BP) agar added with rabbit plasma fibrinogen, incubated at 37 °C for 48 h; *Listeria monocytogenes* on *Listeria* selective agar base (LSAB) added with SR0140E supplement, incubated at 37 °C for 48 h; *Escherichia coli* and *Salmonella* spp., detected on Hektoen enteric agar (HEA), incubated at 37 °C for 24 h. Anaerobiosis occurred in hermetically sealed jars added with the AnaeroGen AN25 system (Oxoid, Milan, Italy).

Cell suspensions of raw milk and pasteurized milk were subjected to plate count for the enumeration of TMM on Skim milk agar (SMA) ISO 6610 [17] incubated aerobically at 30 °C for 72 h; mesophilic rod-shaped LAB on MRS agar, acidified to pH 5.4 with lactic acid (5 mol/L) and incubated anaerobically for 48 h at 30 °C; thermophilic LAB rods were pour plated on whey-based agar medium (WBAM) prepared as described by Settanni et al. [18]; mesophilic and thermophilic coccus-shaped LAB on M17 agar, incubated anaerobically for 48 h at 30 and 44 °C, respectively.

Milk inoculated with each NMSC, curd and cheese samples were analyzed only for TMM and *L. lactis* on SMA and M17 agar, respectively. The last media were incubated as reported above.

All media and supplements were purchased from Biotec, except HEA provided by Microbiol Diagnostici (Uta, Italy). Plates counts were performed in duplicate.

### 2.4. Persistence of Added Strains and Identification of the Survival Indigenous Milk LAB

The persistence of the strains added as starter cultures and their dominance over the indigenous milk LAB resistant to pasteurization process was performed by randomly amplified polymorphic DNA (RAPD)-PCR technique as reported by Alfonzo et al. [19]. The colonies developed from the highest dilutions on the agar media used for LAB counts were randomly picked up, purified by successive sub-culturing, and tested for Gram reaction and catalase activity [20]. The presumptive LAB cultures, grown overnight, were subjected to DNA extraction using the DNA-SORB-B kit (Sacace Biotechnologies Srl, Como, Italy) following the protocol provided by the manufacturer. RAPD-PCR was performed by means of Swift™ MaxPro Thermal Cycler (Esco Health Care Pte. Ltd., Singapore) and the amplicons were separated by electrophoresis. The recognition of the added strains was performed by comparing the polymorphic profiles obtained from the isolates of a given trial with that of the pure culture added using the software Gelcompare II version 6.5 (Applied-Maths, Sin Marten Latem, Belgium).

The identification of the different LAB strains isolated from pasteurized bulk milk before NMSC inoculation was carried out by 16S rRNA gene sequencing as reported by Weisburg et al. [21]. The resulting DNA fragments were purified and sequenced by Eurofins Genomics (Ebersberg, Germany). The sequences were compared with the sequences available in GenBank/EMBL/DDBJ (http://www.ncbi.nlm.nih.gov) and Ez-Taxon-e (http://eztaxon-e.ezbiocloud.net/) databases.

### 2.5. Physicochemical Analyses of Cheeses

Cheeses after 15 d of refrigerate storage were assessed for external and internal color, measured in duplicate by a Minolta Chroma Meter CR300 (Minolta, Osaka, Japan) using the illuminant C; results are expressed as lightness (L*, from 0 = black, to 100 = white), redness (a*, from red = +a, to green = −a), and yellowness (b*, from yellow = +b, to blue = −b), according to the CIE L* a* b* system. Cheese hardness was evaluated with an Instron 5564 tester (Instron, Trezzano sul Naviglio, Milan, Italy) measuring the maximum resistance to compression (compressive stress, N/mm^2^) of samples (2 cm × 2 cm × 2 cm) kept at room temperature (22 °C).

Cheese samples were freeze-dried and analyzed using standard methods of the International Dairy Federation for dry matter (DM) (IDF, 4A:1982) [22], fat (IDF, 5B:1986) [23], protein (N × 6.38) (IDF, 25:1964a) [24], and ash (IDF, 27:1964b) [25] content. In addition, the products of secondary lipid oxidation were assessed in duplicate on freeze-dried samples by determining the thiobarbituric acid-reactive substances (TBARs), expressed as μg malonylaldehyde (MDA)/kg DM, as described by Bonanno et al. [26].

### 2.6. Volatile Organic Compounds

Five grams of the final cheese samples were finely chopped and placed into 25 mL glass vials sealed with silicon septum. The headspace solid phase microextraction SPME (DVB/CAR/PDMS, 50 mm, Supelco) fiber was exposed to the cheese under continuous stirring at 60 °C for 15 min. After sampling, the SPME fiber was thermally desorbed for 1 min through a splitless GC injector at 250 °C. A gas chromatograph (Agilent 6890) equipped with a mass selective detector (Agilent 5975 c) and a DB-624 capillary column (Agilent Technologies, 60 m, 0.25 mm, 1.40 µm) was used for the chromatographic analysis. Chromatographic conditions applied were helium carrier gas at 1 mL/min and an oven temperature program with a 5 min isotherm at 40 °C followed by a linear temperature increase of 5 °C min up to 200 °C, where it was held for 2 min. The MS scan conditions were interface temperature was 230 °C acquisition mass range was 40–400; acquisition mode was scan. The volatile organic compounds were also determined for GPP.

The identification of VOC compounds was performed through the comparison of the MS spectra with commercial library NIST05. The relative proportions of the identified constituents were expressed as percentages obtained by GC-MS peak area normalization with total area of significant peaks. Three replicates of each sample were analyzed.

### 2.7. Sensory Evaluation

The cheeses were also evaluated for their sensory traits. All cheeses were judged by 12 assessor members including 6 men and 6 women (aged between 21–65 years old) familiar with the sensory analysis of cheese. All panelists were specifically trained for cheese attribute evaluation following the ISO 8589 [27] indications. The cheeses were acclimated at about 20 °C for 1 h, cut into cubes (3 cm × 3 cm × 3 cm) and then coded and served in a random order. Twelve descriptive attributes were judged as evaluated by Costa et al. [28]. In particular, for each cheese, the evaluation considered the following aspects: Intensity of odor and aroma, sweet, salt, bitter, acid, fiber, friability, adhesiveness, hardness, humidity, and the overall assessment. Each aspect was scored using a line scale from 0 to 7 (cm) as reported by Faccia et al. [29].

### 2.8. In Vitro Gastrointestinal Digestion

Simulated in vitro human digestion, mimicking physicochemical and biochemical changes of the food in the upper gastrointestinal (GI) tract, was performed (*n* = 3) as reported by Attanzio et al. [30]. Samples of 7.5 g of cheese were added in 20 mL of a buffered pH 6.8 solution simulating saliva [NaCl (0.126 g), KC1 (0.964 g) KSCN (0.189 g), KH_2_PO_4_ (0.655 g), urea (0.200 g), Na_2_SO_4_·10H_2_O (0.763 g), NH_4_Cl (0.178 g), CaCl_2_·2H_2_O (0.228 g), and NaHCO_3_ (0.631 g) in 1 L of distilled water]. The mixture was blended for 3 times × 15 s in a semi-micro stainless-steel blender jar using a Waring blender (Waring, New Hartford, CT, USA) miming the oral phase of digestion. Post-oral samples were then transferred into a bottle, acidified at pH = 2.0 with HCl before porcine pepsine addition (8 mg/mL; 3200–4500 units/mg, Sigma-Aldrich). The bottle was sealed and incubated in a water bath (type M 428-BD, Instruments s.r.l., Bernaggio, Mi, Italy) with shaking (100 rpm), at 37 °C for 2 h, in the dark. Post-gastric digested were brought to a final pH 7.5 adding 200 mM NaH_2_PO_4/_Na_2_HPO_4_ buffer and 5 M NaOH. Then porcine bile extract (2.4 mg/mL, Sigma) and pancreatin from hog pancreas (0.4 mg/mL, Sigma) were added. The bottle was sealed and incubated in the shaking water bath at 37 °C for 2 h, in the dark. The digestion was stopped by ice bath immersion. Post-intestinal digested was centrifuged at 167,000× *g* for 35 min at 4 °C (Beckman Optima TLX ultracentrifuge, Beckman Instruments, Inc., Palo Alto, CA, USA) to separate supernatant (Bioaccesible fraction) from the particulate material. Aliquots of samples from each digestion step were withdrawn and centrifuged at 1500× *g* for 10 min at 4 °C. Supernatants and bioaccesible fraction were brought at pH 2.0 to stabilize polyphenols and stored at −80 °C until analysis.

### 2.9. ABTS ^+^ Radical Cation Decolorization Assay

Radical scavenging activity was evaluated using the ABTS ^+^ radical cation decolorization assay as described by Attanzio et al. [31]. Samples were analyzed in duplicate, at three different dilutions, within the linearity range of the assay. The assay was standardized with the water-soluble vitamin E analog Trolox (Sigma), and results were expressed as μmol Trolox equivalents/g cheese.

### 2.10. Lipid Peroxidation Assay

Pig’s brain microsomes were prepared from a tissue homogenate in 10 mM phosphate buffer saline, pH 7.4 (PBS), by differential centrifugation. Microsomes in PBS (2 mg protein/mL) were pre-incubated for 5 min at 37 °C either in the absence (control) or in the presence of variable amounts of the bioaccessible fraction of cheeses. Lipid oxidation was induced by 20 mM 2,2′-azobis (2-amidino-propane) dihydrochloride] (AAPH, Sigma). Lipid hydroperoxides formation was monitored after reaction with thiobarbituric acid (TBA), as TBA-reactive substances (TBARS). Aliquots (1 mL) of the reaction mixture were added to 2 mL of a solution containing 15% TCA (*v*/*v*), 0.375% TBA (*w*/*v*), 0.25 N HCl, and 0.02% BHT (*w*/*v*), to prevent formation of non-specific TBA-RS and decomposition of AAPH during the subsequent boiling. The mixture was incubated in a boiling water bath for 30 min. After cooling, samples were clarified by centrifugation, and TBA-RS in the supernatant determined at 532 nm. The results are expressed as nmol of MDA equiv/mg of protein, using the molar extinction coefficient of 156,000.

Proteins in microsomal preparations were determined by the Bio Rad colorimetric method [32].

### 2.11. Statistical Analyses

Data of microbiological analyses and physicochemical traits of 15 d cheeses were statistically analyzed using the generalized linear model (GLM) procedure in SAS 9.2 (SAS, 2010) to evaluate the effects of cheese trial (1, 2), treatment (TR) with grape pomace powder (control, experimental), starter culture (NMSC: Mise36, Mise94, Mise169, Mise190) and the interaction TR*NMSC. When a statistically significant effect (*p* ≤ 0.05) of NMSC and TR*NMSC interaction was detected, means were compared using p-values adjusted according to the Tukey–Kramer multiple comparisons test. The results of in vitro digestion of cheeses were made using one-way ANOVA test, with Tukey’s correction for multiple comparisons by Instat-3 statistical software (GraphPad Software Inc., San Diego, CA, USA). Comparison between individual group means was performed by unpaired Student’s *t*-test. In all cases, significance was accepted if the null hypothesis was rejected at the *p* < 0.05.

## 3. Results and Discussion

### 3.1. Acidification Kinetics of Curds

**Figure 1:**
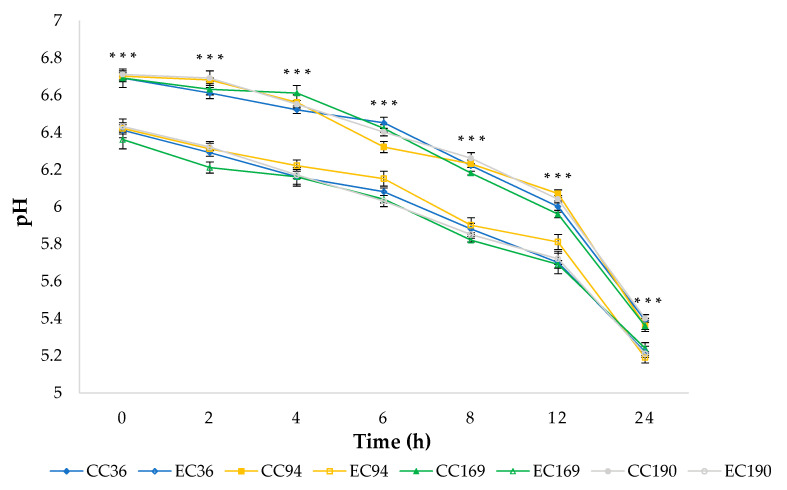
Acidification kinetics of curds. Full symbols: Control curds. Empty symbols: Experimental curds. Results indicate mean values ± SD of four determinations (carried out in duplicate for two independent productions). In comparison with the corresponding result of grape pomace powder (GPP)-enriched curd, values are significant with *** *p* < 0.0001. Abbreviations: CC36, CC94, CC169, and CC190, control curd with *L. lactis* MISE36, MISE94, MISE169, and MISE190, respectively; EC36, EC94, EC169, and EC190, experimental curd with 1% of GPP and *L. lactis* MISE36, MISE94, MISE169, and MISE190, respectively. Bars represent standard deviation of the mean.

The evolution of curd pH is shown in Figure 1. Statistically significant differences were found between control and experimental curds for all strains inoculated. The initial values of pH of control curds were at ca. 6.7, while lower values (ca. 6.4) were registered for the experimental curds. These differences (*p* < 0.0001) were registered until the end of the acidification process.

**Figure 2:**
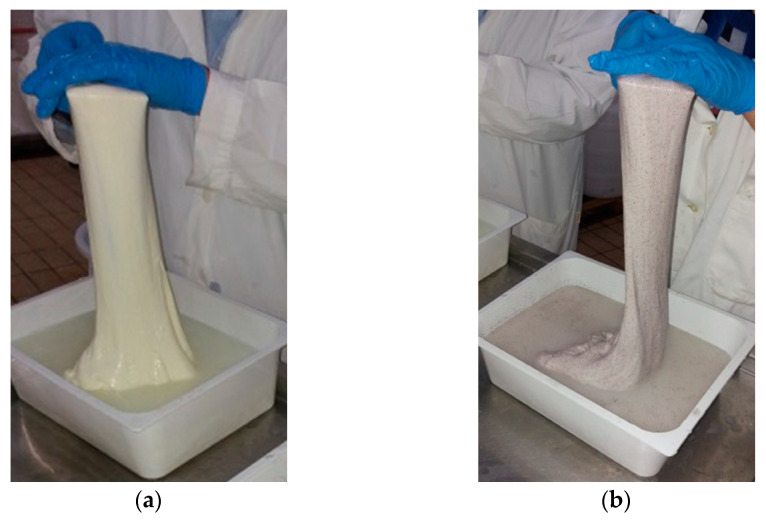
Stretching step of the acidified curds. (**a**) Control cheese production; (**b**) GPP-enriched cheese production.

The lower values of pH in experimental curds are imputable to the presence of organic acids such as tartaric acid, malic acid, and citric acid in GPP [7]. However, all curds reached the value comprised in the range 5.20–5.40, which represents the optimum level of acidity allowing the stretching of the curd [33], about 24 h after curdling (Figure 2).

### 3.2. Microbiological Analyses

The microbiological loads of the samples collected during the cheese-making trials are reported in Table 2. The microbiological analyses of GPP did not evidence the presence of any of the microbial groups object of investigation. The absence of microorganisms in GPP is undoubtedly due to the oven-drying stabilization [34]. Raw ewes’ milk hosted levels of TMM of 6.51 Log CFU/mL, higher than the limit (<10^5^ CFU/mL) established in Europe for raw ewes’ milk for cheese production, with mesophilic rod and coccus LAB at 10^6^ CFU/mL and thermophilic rod and coccus LAB at 10^4^ CFU/mL. The high levels probably depend on the conservation conditions [35]. After pasteurization, TMM, thermophilic rod, and coccus LAB were recorded at 10^3^ CFU/mL, while mesophilic rod and coccus LAB were lower than 10^2^ CFU/mL, showing the ability of the indigenous thermoduric LAB to survive during the thermal treatment [36]. However, the effect of pasteurization depends on the initial cell densities of microorganisms in the raw milk [37] and the temperatures applied [38]. According to Tukey’s test, statistically significant differences were found only for the levels of TMM in curd samples. The levels of TMM were almost superimposable to those of mesophilic coccus LAB in all samples analyzed. After inoculation with each NMSC, all milks showed approximately 7 Log cycles of TMM and almost the same levels of mesophilic coccus LAB, confirming that the inoculums occurred at 10^7^ CFU/mL and the starter LAB cocci dominated the microbial community of cheeses. After coagulation, an increase of about 1 Log cycle was registered for the LAB levels in all control and experimental curds as a consequence of whey draining [39]. The levels of TMM and mesophilic coccus LAB reached values of about 9 log CFU/g in all acidified curds and these levels remained almost constant in control and experimental cheeses soon after production as well as after 15 d of refrigerated storage as previously observed by Gaglio et al. [40,41] during the production of Vastedda della valle del Belìce PDO cheeses. The results highlighted that the addition of 1% (*w*/*w*) of GPP did not influence the fermentation process carried out by the four *L. lactis* (Mise36, Mise94, Mise169, and Mise190) strains used individually.

### 3.3. Persistence of LAB Inoculums

**Figure 3:**
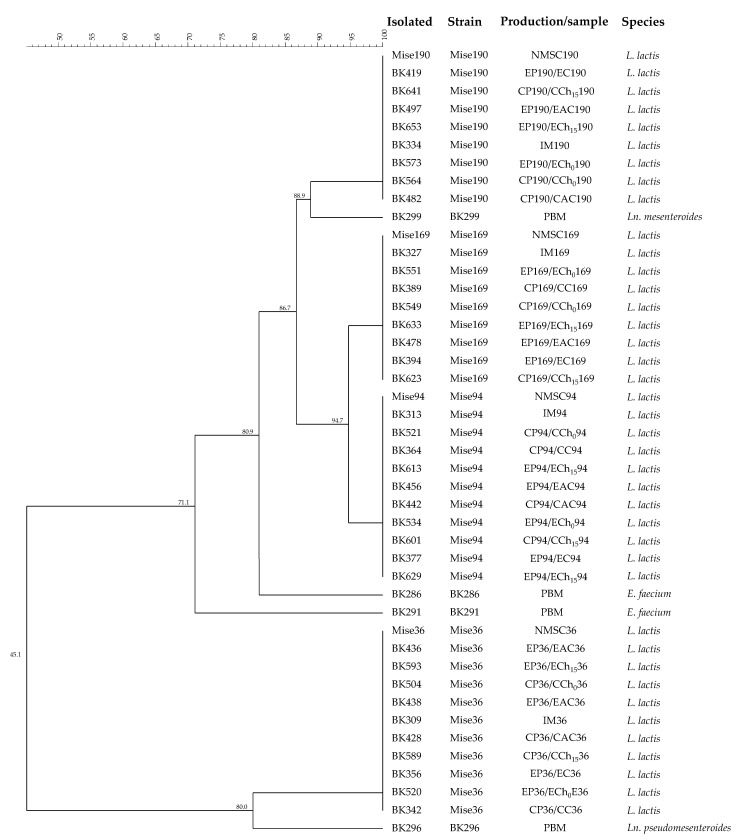
Dendrogram obtained with combined randomly amplified polymorphic DNA (RAPD)-PCR patterns generated with three primers for lactic acid bacteria (LAB) strains isolated during cheese productions. The line at the top indicates percentages of similarity. Abbreviations: *L*., *Lactococcus*; *E.*, *Enterococcus*; *Ln.*, *Leuconostoc*; BK, Biopek dairy factory; CP, control production; EP, experimental production; IM36, IM94, IM169, and IM190, inoculated milk with *L. lactis* MISE36, MISE94, MISE169, and MISE190, respectively; CC36, CC94, CC169, and CC190, control curd with *L. lactis* MISE36, MISE94, MISE169, and MISE190, respectively; EC36, EC94, EC169, and EC190, experimental curd with 1% of GPP and *L. lactis* MISE36, MISE94, MISE169, and MISE190, respectively; CAC36, CAC94, CAC169, and CAC190, control acidified curd with *L. lactis* MISE36, MISE94, MISE169, and MISE190, respectively; EAC36, EAC94, EAC169, and EAC190, experimental acidified curd with 1% of GPP and *L. lactis* MISE36, MISE94, MISE169, and MISE190, respectively; CCh_0_36, CCh_0_94, CCh_0_169, and CCh_0_190, control cheese with *L. lactis* MISE36; MISE94, MISE169, and MISE190, respectively; ECh_0_36, ECh_0_94, ECh_0_169, and ECh_0_190 experimental cheese with 1% of GPP and *L. lactis* MISE36, MISE94, MISE169, and MISE190, respectively; CCh_15_36, CCh_0_94, CCh_15_169, and CCh_15_190, control cheese after 15 d of refrigerate storage with *L. lactis* MISE36; MISE94, MISE169, and MISE190, respectively; ECh_15_36, ECh_15_94, ECh_15_169, and ECh_15_190 experimental cheese after 15 d of refrigerate storage with 1% of GPP and *L. lactis* MISE36, MISE94, MISE169, and MISE190, respectively; PBM, pasteurized bulk milk before inoculum.

Three hundred and ninety-six colonies of presumptive LAB were isolated during cheese making, from pasteurized bulk milk until stored cheeses. After Gram and catalase tests, 360 Gram-positive and catalase-negative cultures were still considered presumptive LAB cultures. The determination of starter persistence by means of RAPD patterns comparison is a common technique to monitor the dynamics of the added strains and their dominance over indigenous bacteria during cheese productions [33,40]. Figure 3 shows the dendrogram with only 44 of the 360 LAB strains analyzed, which are those isolated at least once from the different samples.

Four major RAPD clusters were identified. Each cluster included one *L. lactis* strain used for the preparation of the NMSCs. Four LAB strains (BK286, BK291, BK296, and BK299) collected from pasteurized bulk milk before inoculum were subjected to the 16S rRNA gene sequencing and identified as *Enteroccus faecium* (Ac. No. MW283889–MW283890), *Leuconostoc mesenteroides* (Ac. No. MW283891) and *Leuconostoc pseudomesenteroides* (Ac. No. MW283892). These species are part of the common dairy starter LAB and non-starter LAB cultures [42]. However, these strains were not detected in any of the samples analyzed after milk inoculation with NMSCs, highlighting the dominance of the selected *L. lactis* strains (Mise36, Mise94, Mise169 and Mise190) over the indigenous thermoduric milk LAB (Figure 2).

### 3.4. Physicochemical Analyses of Cheeses

The physicochemical parameters of the final cheeses are reported in the Table 3. As expected, the inclusion of GPP modified markedly both external and internal color indexes. Indeed, the experimental cheeses acquired an evident pink color, as indicated by the strong redness increase, to which corresponded the reduction of lightness and yellowness, whereas the indexes recorded on control cheeses were in line with those reported for ovine stretched cheeses by Todaro et al. [43].

The chemical composition of cheeses was affected by both GPP inclusion and the starter cultures. GPP are characterized by lower lipid content than milk determining a decrease of fat and, consequently, an increase of protein in GPP-enriched cheeses. The increase of ash can be referred to the higher ash level of GPP compared to milk. Similar trends for lipid, protein, and ash concentrations were observed by Marchiani et al. [7] in cheeses fortified with GPP of different origin. Regarding the starter strains, a different behavior was observed for *L. lactis* MISE94 and MISE190 that in experimental cheeses induced a stronger fat decrease, which was accompanied by a protein increase, suggesting a more intense recourse to fatty acids to sustain their energy metabolism [44]. Nevertheless, the levels recorded for each chemical component were within the ranges observed in other investigations for Vastedda cheeses [43].

GPP inclusion was also responsible for the hardness increase of the cheese paste, evaluated as resistance to compression, presumably due to the effect of GPP in increasing DM or lowering fat content in experimental cheeses; however, in accordance to the results of sensory evaluation, the difference for hardness was almost negligible when the strain *L. lactis* MISE36 strain was used, an aspect that can be referred to the lower difference in fat content together with a similar DM level between the corresponding control and GPP-enriched cheeses.

Moreover, higher TBARS values were observed in experimental rather than control cheeses, indicating that the presence of GPP did not inhibit the lipid oxidation, despite their interesting content in phenolic compounds with antioxidant activity [7,44]. Since the experimental cheeses were lower in fat, this higher presence of oxidation products could be linked to their enrichment with the polyunsaturated fatty acids that characterizes the lipid profile of GPP [44] and are more sensitive to oxidation.

### 3.5. Volatile Organic Compounds

Volatile profiles are generated by complex biochemical processes derived from the hydrolysis or metabolism of carbohydrates, proteins, and fats, along with compounds added during processing or directly from the milk due to the activity of LAB [45]. VOCs were identified in cheese samples and GPP by SPME-GC/MS technique (Table 4).

A total of 21 VOCs were detected in GPP, whereas 21 and 14 VOCs were identified in cheese samples with and without GPP addition, respectively. Volatile compounds belonging to alkanes, aldehydes, monoterpenes, esters, acids, and ketones were identified in all cheese samples. Acetic acid and 2-hydroxy4-methyl pentanoic acid were the most abundant acids detected in cheese sample without GPP addition. Acetic acid may be produced by carbohydrate catabolism by LAB, 2-hydroxy-4-methyl pentanoic acid is formed enzymatically from the corresponding amino acid (L-leucine) [46]. In addition, the free fatty acids (FAA) identified in this study were hexanoic, butyric, and 3-methyl-butanoic acid. Butyric and acetic acids were the main acids identified in cheese sample with GPP addition. FFA contribute to the formation of cheese flavor directly and as precursors of ketones, alcohols, aldehydes, and esters [47,48]. Similar FFA profiles were also observed in other cheeses produced from sheep’s milk [41,46]. Two aldehydes (hexenal and heptanal) and one ester (octanoinc acid ethyl ester) were detected in the experimental cheese samples. No ester was identified in the control cheeses. Aldehydes and esters are poorly represented in Vastedda cheeses [49], probably because they are oxidized to acids or reduced to n-alkanols due to the enzymatic activities of microorganisms [50]. Isoamyl alcohol, 2-butanol, 2-pentanol, and phenyl-ethyl-alcol were the only alcohols detected in cheese samples with isoamyl alcohol being the major alcohol present in all cheeses. A total of eight hydrocarbons and terpenes were emitted from the cheeses. Terpenes and hydrocarbons originated from the secondary metabolism of plants [51], therefore they can be rapidly transferred into milk fat by forages or other substances added to cheese. In light of the GPP VOCs profile, we can assess that the addition of GPP contributed to increase the amount of isoamyl alcohol and to determine the presence of D-limonene and 2-phenylethanol in the experimental cheeses.

### 3.6. Sensory Test

**Figure 4:**
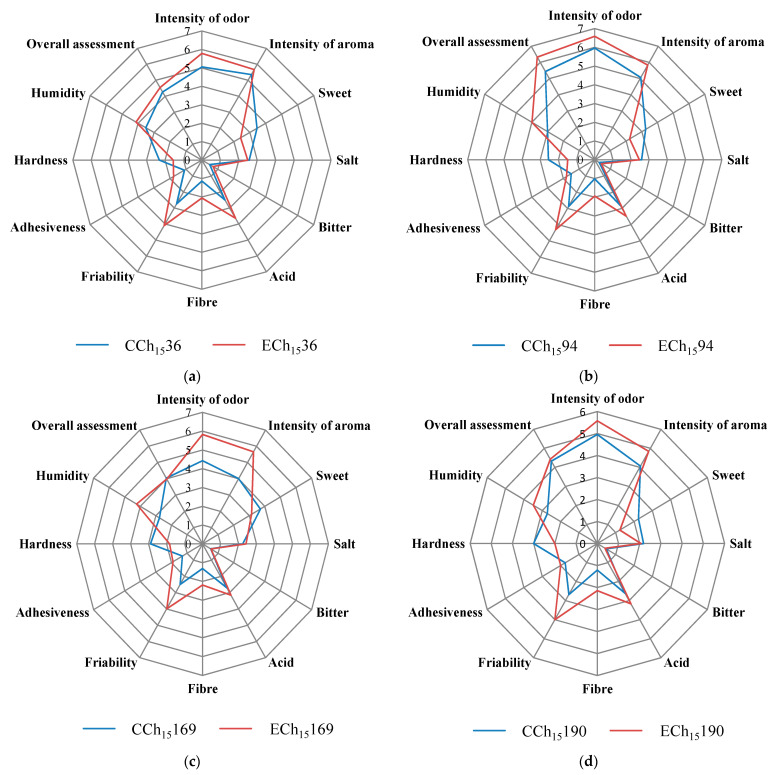
Spider diagrams corresponding to the descriptive sensory analysis of cheeses. (**a**) CCh_15_36, control cheese after 15 days of refrigerate storage with *L. lactis* MISE36; ECh_15_36, experimental cheese after 15 days of refrigerate storage with *L. lactis* MISE36 + 1% of GPP; (**b**) CCh_15_94, control cheese after 15 days of refrigerate storage with *L. lactis* MISE94; ECh_15_94, experimental cheese after 15 days of refrigerate storage with *L. lactis* MISE94 + 1% of GPP; (**c**) CCh_15_169, control cheese after 15 days of refrigerate storage with *L. lactis* MISE169; ECh_15_169, experimental cheese after 15 days of refrigerate storage with *L. lactis* MISE169+ 1% of GPP; (**d**) CCh_15_190, control cheese after 15 days of refrigerate storage with *L. lactis* MISE190; ECh_15_16, experimental cheese after 15 days of refrigerate storage with *L. lactis* MISE190+ 1% of GPP.

All cheeses, after 15 days of refrigerated storage, were subjected to the sensory analysis and the results comparing control and experimental samples per each *L. lactis* strain are reported in Figure 4. The addition of GPP modified the sensory attributes of cheeses. Generally, the addition of fibers or by-products exert a strong effect on the sensory parameters of dairy products [37,52]. In this study, except for sweet and bitter that scored similar values in all trials, all other attributes evaluated were consistently influenced by the addition of GPP. In detail, the addition of GPP increased odor and aroma intensity, acid perception, fiber sensation, friability, adhesiveness, and humidity while influenced negatively sweet and hardness attributes. Similar results were observed by Costa et al. [28] who tested white and red wine grape pomace to fortify bovine Primosale cheese. The selected LAB added as starter cultures determined different effects on the sensory attributes of cheeses (Figure 4a–d). The cheeses produced with the strain Mise94 (with and without GPP) were mostly appreciated by the judges.

### 3.7. Functional Properties

GPP is a rich source of polyphenols, which are the most abundant antioxidants in the human diet with potential health benefits [53]. The antioxidant capacity of GPP-added cheese was investigated only for the cheeses mostly appreciated by the judges; for this reason, ECh_15_94 and CCh_15_94 were analyzed through ABTS ^+^ radical cation decolorization assay. Both cheeses were submitted to the in vitro gastro-intestinal digestion and the ability of reduction of the radical was evaluated from the different digestion steps (Table 5).

Post-oral fraction of the control cheese showed reducing activity that did not significantly (*p* > 0.05) vary after gastric digestion (0.202 ± 0.012 µmol TE/g). Milk components, mainly high molecular weight casein, are considered responsible for the antioxidant capacity of the dairy products [54]. Moreover, it was reported that pepsin digestion does not increase radical scavenging activity of purified casein [55]. After intestinal digestion, the antioxidant activity of control CCh_15_94 cheese was about 50% higher than that measured after gastric phase, possibly due to the antioxidant fat-soluble vitamins released in the bile salts micelles or to the amino acids with reducing ability [56,57]. When GPP-added cheese (ECh_15_94) was submitted to the in vitro digestion, the antioxidant potential of the post-oral fraction was not recorded different from that of the relevant fraction of control cheese (*p* > 0.05), indicating that dilution and homogenization in simulated saliva fluid did not cause polyphenols release (Table 5). Interestingly, a net rise of the reducing activity (+41%) was measured in the samples after gastric digestion. As reported by Tagliazucchi et al. [58], casein digestion by pepsin can affect the binding with incorporated polyphenols, resulting in their solubilization. The antioxidant activity of ECh_15_94 cheese increased also after the intestinal step (Table 5). Finally, all reducing components released through the digestion of both cheeses were solubilized in the bioaccessible fraction, i.e., the soluble fraction of the digesta available for absorption (Table 5). Overall, during the digestion process, with the exception of the oral phase, all fractions obtained from the digestion of the GPP containing cheese showed a much higher antioxidant capacity than GPP-free cheese. The reducing activity of the bioaccessible fraction of ECh_15_94 accounted for 175% of that measured in the corresponding fraction of CCh_15_94. Our results demonstrated the important contribution of the polyphenols of GPP to the antioxidant capacity of the cheese and highlighted the functional potential of the fortified product.

**Figure 5:**
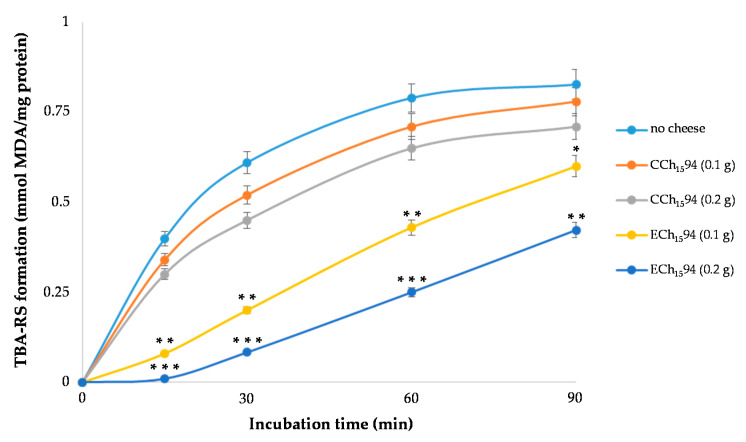
Time-course of thiobarbituric acid-reactive substances (TBA-RS) formation during the AAPH-induced microsomal oxidation, either in the absence (control) or in the presence of bioaccessible fraction obtained after in vitro digestion of cheeses. Microsomes, at 2 mg of protein per mL of reaction mixture, were submitted to peroxidation by AAPH as reported in the Materials and Methods. Each value is the mean ± SD of three determinations performed in duplicate. In comparison with the corresponding amount of GPP-free cheese, values are significant with * *p* < 0.05; ** *p* < 0.01; *** *p* < 0.0001. Abbreviations: CCh_15_94, control cheese after 15 d of refrigerate storage with *L. lactis* MISE94; ECh_15_94, experimental cheese after 15 d of refrigerate storage with 1% of GPP and *L. lactis* MISE94.

Unregulated food lipids oxidation during digestion can lead to a postprandial oxidative stress condition, which negatively affects the human health [59]. Consequently, antioxidants contained in foods that are able to react with and detoxify lypoperoxyl radicals can be considered of interest to reduce the oxidative phenomena during digestion. Peroxyl radical scavenger activity of the bioaccessible fractions of the cheeses was compared against lipid oxidation of pig brain microsomes. When lipid peroxidation was induced in microsomes by AAPH in the absence of cheese, TBA-RS production started immediately reaching a maximum at 90 min incubation (Figure 4). Bioaccessible fraction from 0.1 g or 0.2 g of CCh_15_94 slightly slowed down TBA-RS formation in a dose-dependent manner. Interestingly, bioaccessible fraction from GPP-enriched cheese (ECh_15_94) caused a stronger dose-dependent inhibition of the lipid peroxidation, evidenced by the net delay in the initial TBA-RS formation and a higher reduction of TBA-RS production throughout the incubation period (Figure 5).

These results demonstrated that GPP caused 60% increase of the lipoperoxyl radical scavenger capacity of the cheese measured after digestion, conferring a potential healthy value against postprandial oxidative stress.

## 4. Conclusions

This study provided, for the first time, an extended analysis of the microbiological, physicochemical, sensory, and functional aspects of GPP-enriched ovine cheese. The addition of GPP did not alter the microbiological parameters during the fermentation carried out with four strains of *L. lactis* used as single inoculums. The chemical composition of cheeses was affected by GPP addition. GPP-enriched cheeses were characterized by lower fat content, higher protein content, and higher values of secondary lipid oxidation. The differences in VOCs detected between control and experimental cheeses are a direct consequence of GPP addition. Sensory evaluation indicated that the GPP-enriched cheeses were characterized by a general appreciation by judges and, in particular, the higher values of overall acceptance was detected for the cheeses produced with the strain MISE94. In particular, from the functional point of view, the cheeses enriched with GPP, submitted to simulated human digestion, showed an increased antioxidant activity and lipoperoxyl radical scavenger capacity. Degradation of dairy matrix appears a requisite to release the incorporated polyphenols in GPP-enriched cheese and increase the reducing activity of the intestinal digesta. However, due to the importance of using mixed strain starters to ensure the fermentation process, further studies are necessary to validate this technology for industrial applications.

## Figures and Tables

**Table 1:** Sampling points ^a^ and analyses performed during cheese production.

Analyses	Sampling Points										
	BM	PBM	IM	Curd t_0_		Acidified Curds		Cheese at t_0_		Cheese at 15 d	
				Ctr	Exp	Ctr	Exp	Ctr	Exp	Ctr	Exp
pH	■	■		■	■	■	■				
plate counts	■	■	■	■	■	■	■	■	■	■	■
molecular typing		■	■	■	■	■	■	■	■	■	■
physical aspects										■	■
chemical composition										■	■
VOCs										■	■
sensory tests										■	■
functional properties										■	■

^a^ Two samples were analyzed for each production. Abbreviations: BM, bulk milk; PBM, pasteurized bulk milk before inoculum; IM, inoculated milk with Natural Milk starter cultures (NMSCs); Ctr, control; Exp, experimental; VOCs, volatile organic compounds.

**Table 2:** Microbial evolution ^a^ during experimental cheese productions.

		Treatment (TR)		Natural Milk Starter Culture (NMSC)					Significance *p*<		
				MISE36	MISE94	MISE169	MISE190	SEM	TR	NMSC	TR*NMSC
Inoculated milk	TMM			7.23 ab	7.33 a	6.94 ab	7.08 b	0.088		0.0442	
	MCLAB			7.30	6.95	7.04	7.18	0.14		0.3546	
Curd t_0_	TMM	Ctr	7.93	8.12	7.98	7.83	7.77	0.10	0.0227	0.0096	0.7364
		Exp	7.75	7.99	7.71	7.61	7.71				
		Tot		8.06 a	7.84 ab	7.72 b	7.74 b				
	MCLAB	Ctr	7.77	7.89	7.81	7.74	7.63	0.13	0.7777	0.3440	0.7577
		Exp	7.74	7.78	7.89	7.60	7.70				
		Tot		7.83	7.85	7.67	7.66				
Acidified curd	TMM	Ctr	9.13	9.01	9.23	9.15	9.14	0.12	0.7408	0.2539	0.8490
		Exp	9.16	9.07	9.33	9.20	9.04				
		Tot		9.04	9.28	9.18	9.09				
	MCLAB	Ctr	9.44	9.47	9.35	9.44	9.49	0.10	0.5935	0.3304	0.1127
		Exp	9.48	9.38	9.53	9.69	9.30				
		Tot		9.42	9.44	9.57	9.39				
Cheese at t_0_	TMM	Ctr	8.66	8.66	8.63	8.69	8.66	0.13	0.3307	0.9658	0.9431
		Exp	8.57	8.57	8.55	8.50	8.64				
		Tot		8.62	8.59	8.59	8.65				
	MCLAB	Ctr	8.62	8.70	8.52	8.77	8.49	0.13	0.8256	0.4783	0.8644
		Exp	8.60	8.61	8.56	8.65	8.57				
		Tot		8.65	8.54	8.71	8.53				
Cheese at t_15_	TMM	Ctr	8.73	8.62	8.98	8.60	8.71	0.075	0.8795	0.0074	0.2972
		Exp	8.74	8.72	8.81	8.63	8.79				
		Tot		8.67 b	8.89 a	8.62 b	8.75 ab				
	MCLAB	Ctr	8.76	8.70	8.89	8.69	8.76	0.071	0.8147	0.5271	0.3911
		Exp	8.75	8.77	8.74	8.75	8.74				
		Tot		8.73	8.82	8.72	8.75				

^a^ Units are log CFU/mL for liquid samples and log CFU/g for solid samples. Results indicate mean values of four plate counts (carried out in duplicate for two independent productions). On the row: a, b = *p* < 0.05. Abbreviations: SEM, standard error of means of interaction; TMM, total mesophilic microorganisms; MCLAB, mesophilic coccus lactic acid bacteria; Ctr, control; Exp, experimental; Tot, total.

**Table 3:** Physicochemical traits of cheeses after 15 d of refrigerated storage.

		Treatment (TR)		Natural Milk Starter Culture (NMSC)					Significance *p*<		
				MISE 36	MISE 94	MISE 169	MISE 190	SEM	TR	NMSC	TR*NMSC
External colour	lightness (L*)	Ctr	82.47	82.27	83.43	80.33	83.86	2.96	<0.0001	0.4523	0.5840
		Exp	51.60	53.91	47.51	49.99	54.98				
		Tot		68.09	65.47	65.16	69.42				
	redness (a*)	Ctr	−5.35	−5.21	−5.27	−5.64	−5.29	0.30	<0.0001	0.6749	0.3080
		Exp	4.90	4.52	5.25	5.18	4.65				
		Tot		−0.35	−0.01	−0.23	−0.32				
	yellowness (*)	Ctr	18.10	17.77	18.31	18.80	17.54	0.63	<0.0001	0.3406	0.8874
		Exp	4.31	4.59	4.32	4.78	3.57				
		Tot		11.18	11.31	11.79	10.55				
Internal colour	lightness (L*)	Ctr	85.02	84.19	86.66	83.59	85.66	1.71	<0.0001	0.7484	0.1583
		Exp	56.49	59.09	53.24	56.99	56.65				
		Tot		71.64	69.95	70.29	71.15				
	redness (a*)	Ctr	−2.87	−2.92	−2.81	−2.85	−2.91	0.35	<0.0001	0.6809	0.7180
		Exp	5.19	4.88	5.44	4.91	5.54				
		Tot		0.98	1.31	1.03	1.32				
	yellowness (*)	Ctr	10.68	10.77	10.63	10.42	10.92	0.17	<0.0001	0.5230	0.0896
		Exp	3.61	3.76	3.38	3.90	3.42				
		Tot		7.26	7.00	7.16	7.17				
Hardness, N/mm^2^		Ctr	0.44	0.50 a	0.40 Bab	0.52 Ba	0.36 Bb	0.021	<0.0001	0.0059	0.0044
		Exp	0.64	0.58 b	0.67 Aab	0.69 Aa	0.64 Aab				
		Tot		0.54 b	0.53 b	0.61 a	0.50 b				
Chemical composition	Dry matter (DM), %	Ctr	55.34	55.77 a	55.13 b	54.24 Bc	56.21 Aa	0.13	<0.0001	0.0007	<0.0001
		Exp	55.85	55.94 b	55.42 bc	56.82 Aa	55.24 Bc				
		Tot		55.85 a	55.27 b	55.53 ab	55.72 a				
	Ash, % DM	Ctr	5.16	5.40	4.81	5.47	4.96	0.12	0.0015	0.0007	0.2336
		Exp	5.47	5.74	5.37	5.53	5.25				
		Tot		5.57 a	5.09 b	5.50 a	5.11 b				
	Protein, % DM	Ctr	46.01	45.18 b	44.42 Bb	50.17 a	44.28 Bb	0.74	0.0020	0.0007	0.0013
		Exp	47.94	45.81	48.99 A	48.41	48.54 A				
		Tot		45.49 b	46.70 b	49.29 a	46.41 b				
	Fat, % DM	Ctr	46.41	47.40 Ab	48.54 Aa	41.90 c	47.78 Ab	0.12	<0.0001	<0.0001	<0.0001
		Exp	42.42	44.08 Ba	40.83 Bd	41.96 c	42.82 Bb				
		Tot		45.74 a	44.69 c	41.93 d	45.30 b				
TBARS, μg MDA/kg DM		Ctr	18.74	17.57	20.92	19.54	16.94	3.65	<0.0461	0.9722	0.7790
		Exp	24.82	24.31	22.83	25.75	26.41				
		Tot		20.94	21.88	22.65	21.67				

On the row: a, b, c, d = *p* < 0.05; on the column: A, B = *p* < 0.05. Abbreviations: SEM, standard error of means of interaction; TBARs, thiobarbituric acid-reactive substances; MDA, malonylaldehyde; Ctr, control; Exp, experimental; Tot, total.

**Table 4:** Volatile organic compounds emitted from cheeses.

Chemical Compounds ^a^	Samples								
	GPP	CCh_15_36	ECh_15_36	CCh_15_94	ECh_15_94	CCh_15_169	ECh_15_169	CCh_15_190	ECh_15_190
Acids									
Acetic acid	n.d.	9.5	16.2	14.1	14.9	9.2	11.8	18.7	10.4
Butanoic acid	n.d.	9.2	7.5	10.9	7.4	5.7	5.7	9.0	6.0
4-Hydroxybutanoic acid	4.1	n.d.	n.d.	n.d.	n.d.	n.d.	n.d.	n.d.	n.d.
Hexanoic acid	1.6	5.9	5.0	8.2	4.3	4.3	4.0	7.1	4.3
Pentanoinc acid-2-hydroxy-4-methyl	n.d.	16.0	2.5	12.2	7.2	8.0	5.2	13.0	4.7
3-Methylbutanoic acid	n.d.	n.d.	0.5	n.d.	0.3	n.d.	0.2	n.d.	0.4
Nonanoic acid	3.4	n.d.	n.d.	n.d.	n.d.	n.d.	n.d.	n.d.	n.d.
Ketons									
2-Pentanone	n.d.	2.8	0.6	2.4	0.7	1.8	0.6	2.3	0.5
2-Heptanone	n.d.	2.0	0.5	1.9	0.7	2.2	0.6	2.4	0.5
p-Phenylacetophenone	4.2	n.d.	n.d.	n.d.	n.d.	n.d.	n.d.	n.d.	n.d.
Alcohol									
Isoamyl alcohol	4.9	23.7	42.3	27.8	50.0	44.3	51.6	21.5	59.9
2-Pentanol	n.d.	n.d.	0.4	n.d.	0.2	n.d.	0.2	n.d.	0.8
2-Butanol	n.d.	1.3	0.5	1.4	0.5	1.7	0.6	2.3	0.2
2-Phenylethanol	11.3	n.d.	2.6	n.d.	2.3	n.d.	3.9	n.d.	2.7
Hydrocarbons									
Hexane 2-methyl	n.d.	n.d.	0.3	n.d.	0.5	n.d.	0.3	n.d.	0.3
Heptane 2,4-dimethyl	3.2	12.2	3.4	5.6	3.2	9.5	4.6	8.5	5.1
Octane 4-methyl	n.d.	4.4	0.5	1.8	0.5	3.6	0.6	2.5	0.3
Nonane	2.2	n.d.	0.4	n.d.	0.5	n.d.	0.7	n.d.	0.4
Nonane 2,5-methyl	2.3	n.d.	n.d.	n.d.	n.d.	n.d.	n.d.	n.d.	n.d.
Decane	1.8	n.d.	0.4	n.d.	0.3	n.d.	0.5	n.d.	0.2
Dodecane	2.3	n.d.	n.d.	n.d.	n.d.	n.d.	n.d.	n.d.	n.d.
Hexadecane	1.7	n.d.	n.d.	n.d.	n.d.	n.d.	n.d.	n.d.	n.d.
Aldeyde									
Hexanal	3.2	2.6	0.2	1.7	0.4	2.0	0.2	2.8	0.2
Heptanal	n.d.	1.9	0.5	1.9	0.4	1.8	0.1	2.5	0.2
Nonanal	1.7	n.d.	n.d.	n.d.	n.d.	n.d.	n.d.	n.d.	n.d.
Monoterpene									
Phellandrene	n.d.	1.8	n.d.	2.5	n.d.	1.9	n.d.	2.4	n.d.
D-Limonene	6.3	n.d.	12.7	n.d.	2.1	n.d.	5.0	n.d.	0.5
α-Pinene	2.1	6.7	2.6	7.6	3.1	4.1	2.6	5.0	1.9
Carene	1.5	n.d.	n.d.	n.d.	n.d.	n.d.	n.d.	n.d.	n.d.
Esters									
Octanoinc acid, ethyl ester	9.6	n.d.	0.4	n.d.	0.7	n.d.	0.7	n.d.	0.6
Butanedioic acid, diethyl ester	2.2	n.d.	n.d.	n.d.	n.d.	n.d.	n.d.	n.d.	n.d.
Decanoic acid, ethyl ester	9.7	n.d.	n.d.	n.d.	n.d.	n.d.	n.d.	n.d.	n.d.
Diol									
2,3-Butanediol	20.6	n.d.	n.d.	n.d.	n.d.	n.d.	n.d.	n.d.	n.d.

^a^ Data are means percentage of three replicate expressed as (peak area of each compound/total area of significant peaks) × 100. Abbreviations: n.d., not detecteble. Abbreviations: GPP, grape pomace powder; CCh_15_36, CCh_15_94, CCh_15_169 and CCh_15_190, control cheese after 15 d of refrigerate storage with *L. lactis* MISE36; MISE94, MISE169 and MISE190, respectively; ECh_15_36, ECh_15_94, ECh_15_169 and ECh_15_190 experimental cheese after 15 d of refrigerate storage with 1% of GPP and *L. lactis* MISE36, MISE94, MISE169 and MISE190, respectively.

**Table 5:** Antioxidant activity of in vitro digested cheeses measured by ABTS assay.

Digestion Step	CCh_15_94	ECh_15_94
	µmol TE/g	
Post-Oral	0.192 ± 0.011 A	0.215 ± 0.012 A
Post-Gastric	0.202 ± 0.012 A	0.304 ± 0.014 Ba
Post-Intestinal	0.317 ± 0.015 B	0.557 ± 0.022 Cb
Bioaccesible fraction	0.320 ± 0.012 B	0.556 ± 0.023 Cb

Values are the mean ± SD of three separate experiments in duplicate. On the row: a = *p* < 0.05 and b = *p* < 0.01 (Student’s *t*-test); on the column: A, B, C = *p* < 0.05 (Anova one-way followed by Tukey’s test). Abbreviations: CCh_15_94, control cheese after 15 d of refrigerate storage with *L. lactis* MISE94; ECh_15_94, experimental cheese after 15 d of refrigerate storage with 1% of GPP and *L. lactis* MISE94.

## Data Availability

All data included in this study are available upon request by contacting the corresponding author.
